# Targeted mutations in *myostatin* by zinc-finger nucleases result in double-muscled phenotype in Meishan pigs

**DOI:** 10.1038/srep14435

**Published:** 2015-09-24

**Authors:** Lili Qian, Maoxue Tang, Jinzeng Yang, Qingqing Wang, Chunbo Cai, Shengwang Jiang, Hegang Li, Ke Jiang, Pengfei Gao, Dezun Ma, Yaoxing Chen, Xiaorong An, Kui Li, Wentao Cui

**Affiliations:** 1 Institute of Animal Sciences, Chinese Academy of Agricultural Sciences, Beijing 100193, P.R. China; 2 State Key Laboratory of Agrobiotechnology, China Agricultural University, Beijing 100193, P.R. China; 3 Dept of Human Nutrition, Food and Animal Sciences, University of Hawaii at Manoa, Honolulu, Hawaii 96822, USA; 4 Institute of Animal Sciences, Qingdao, 266100, P.R. China; 5 College of Animal Medicine, China Agricultural University, Beijing, 100193, P.R. China

## Abstract

Myostatin (MSTN) is a dominant inhibitor of skeletal muscle development and growth. Mutations in *MSTN* gene can lead to muscle hypertrophy or double-muscled (DM) phenotype in cattle, sheep, dog and human. However, there has not been reported significant muscle phenotypes in pigs in association with *MSTN* mutations. Pigs are an important source of meat production, as well as serve as a preferred animal model for the studies of human disease. To study the impacts of *MSTN* mutations on skeletal muscle growth in pigs, we generated *MSTN*-mutant Meishan pigs with no marker gene via zinc finger nucleases (ZFN) technology. The *MSTN-*mutant pigs developed and grew normally, had increased muscle mass with decreased fat accumulation compared with wild type pigs, and homozygote *MSTN* mutant (*MSTN*^−/−^) pigs had apparent DM phenotype, and individual muscle mass increased by 100% over their wild-type controls (*MSTN*^+/+^) at eight months of age as a result of myofiber hyperplasia. Interestingly, 20% *MSTN-*mutant pigs had one extra thoracic vertebra. The *MSTN-*mutant pigs will not only offer a way of fast genetic improvement of lean meat for local fat-type indigenous pig breeds, but also serve as an important large animal model for biomedical studies of musculoskeletal formation, development and diseases.

*Myostatin* (*MSTN*), also known as growth and differentiation factor-8 (GDF-8), is a member of the transforming growth factor-β superfamily, containing three exons and two introns^1^,^2^. Like other TGF-β family members, MSTN is synthesized as a precursor protein that undergoes proteolytic processing at a dibasic site, located in the front end of exon 3, to generate an N-terminal propeptide and a disulfide linked C-terminal dimer, which is the biologically active molecule^3^,^4^,^5^. Experiments with *MSTN-*knockout mice and the *in vivo* inhibition of MSTN expression by antagonists demonstrated that MSTN plays a negative regulatory role in muscle development^6^,^7^,^8^,^9^,^10^,^11^. It is not surprising that great attention has been focused on MSTN inhibition for increasing lean tissue mass. *MSTN-*knockout mice have a remarkable increase in muscle mass and significant decrease in fat compared to their corresponding wild-type littermates^6^,^12^. The DM cattle caused by natural mutations of *MSTN* loss-of-function have very strong skeletal muscle and contain much less fat^13^,^14^,^15^. Genetic manipulations of myostatin gene or the use of natural *MSTN* mutations for livestock meat production have great potentials to increase feed efficiency and healthy food supplies^16^. Besides its applications in animal agriculture, MSTN inhibition has been a target of medical treatments for various human diseases. Approaches that cause changes in myostatin expression and functions can be used to improve poor nutritional status of muscle, to treat muscular dystrophy or atrophy, and chronic diseases-related cachexia^17^,^18^,^19^. MSTN is also directly or indirectly involved in regulation of fat and glucose metabolism^20^,^21^,^22^,^23^,^24^. It is thus that inhibition of MSTN function can potentially be used as a treatment for obesity and diabetes.

Although amino acid sequences of MSTN are highly conserved across species, and the DM phenotype caused by *MSTN* naturally occurring mutations has been observed in beef cattle such as Belgian Blue and Piedmontese^1^,^2^, sheep^25^, dog^26^, and human^27^, there has been no reports on natural mutations or engineered *MSTN* mutations with loss of MSTN function and dramatic muscular phenotypes in pigs. It remains unknown whether pigs with *MSTN* loss-of-function mutations can survive and show DM phenotype. Pigs not only serve as a major livestock animal source of meat production, but also are used as a large animal model for studying human metabolism and physiology because of the similarity in organ sizes^28^,^29^. Given the size of pig skeletal muscles in the total body mass, results in skeletal muscle mass improvement in pigs can provide much comparable evidences for human studies. Therefore, to study the impact of porcine *MSTN* mutations is very important in agricultural and medical fields.

Meishan (Ms) pigs are a locally famous breed in China, and are well known for their high prolificacy and early sexual maturity, but the breed has a high percentage of carcass fat and poor feed efficiency^30^. These unique qualities make Ms pig a suitable model to test the effects of *MSTN* mutations on muscle growth and body composition.

Recent advancement in genetic manipulation techniques has made it possible to successfully target a gene with high efficiency. Zinc finger nucleases (ZFN) technology overcomes the limitations of embryonic stem cell technology, and allows us to modify the genome of domestic animals with precision and high efficiency^31^,^32^ in combination with somatic cell nucleus transfer (SCNT).

In this study, we successfully generated marker gene-free Ms pigs containing *MSTN*-null mutations by ZFN and SCNT. And the *MSTN*-null Ms pigs had apparent DM phenotype, with greater lean yield and lower fat mass. In addition, we observed that approximately 20% of *MSTN*-mutant pigs contained one more extra thoracic vertebra than that in wild-type Ms pigs. This phenomenon has never been reported in other *MSTN-*mutant animals. These newly generated *MSTN*-mutant Meishan pigs will not only become a significant demonstration of genetic improvement of indigenous fat-type pig breeds for meat production, but also serve as an important large animal model for studying musculoskeletal formation, development, and diseases in the biomedical field.

## Results

### Production of *MSTN* mutant Meishan pigs by ZFN technology

**Figure 1:**
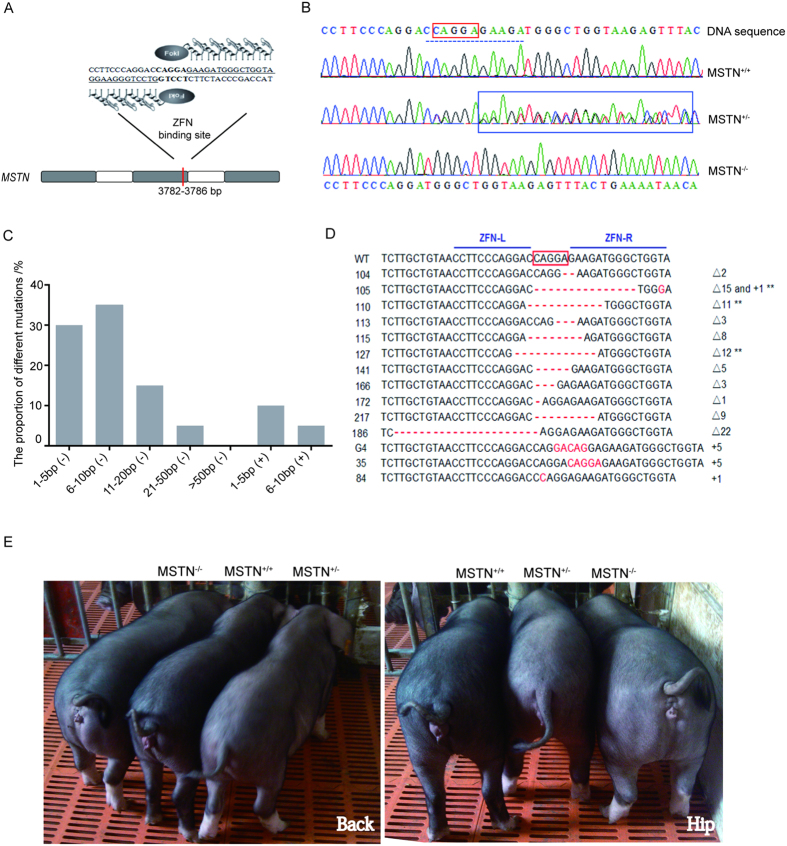
Target disruption of *MSTN* in Meishan (Ms) pigs via ZFNs and photos of *MSTN* mutant Ms pigs. (**A**) Diagram of engineered ZFNs binding to *MSTN* exon 2. Red bar: ZFN-targeted site; gray box: exons; white box: introns. (**B**) Sequencing profiles of wide-type, hemizygous, and homozygous *MSTN* cell clones. Red box: ZFN-targeted site; dotted line: base deletions in homozygous *MSTN* modification. Please note that there are double peaks next to or near the ZFN cleavage site in heterozygous *MSTN* cell clone (see blue box). (**C**) Distribution of different *MSTN* mutation types. Of the 80 mutant cell colonies analyzed, the most dominant mutation types are short-fragment (1–10 bp) deletions. Vertical axis represents the percentage of each mutant type. Horizontal axis represents the sizes of ZFN-mediated fragment deletion (−) and insertion (+). (**D**) Sequences of ZFN-mediated disruption of *MSTN* gene in mutant cell lines. Deletions and insertions are indicated by red dashes and red letters, respectively. Sizes of base insertions (+) or base deletions (Δ) are indicated to the right of each mutant allele. Cell colony #172 is a *MSTN* homozygous mutant that contains deletion of one basepair. However, there is a possibility that alleles with a large deletion might not be detected. **Indicates that cloned live piglets were produced from these cell lines. (**E**) Photos of representative of *MSTN*^−/−^, *MSTN*^+/−^ and *MSTN*^+/+^ pigs (4-month old) generated via breeding F1 heterozygous pigs. Note that *MSTN*^−/−^ pigs show wider back, fuller rump and thicker limbs compared with *MSTN*^+/−^ and *MSTN*^+/+^ pigs.

ZFN plasmid pairs PZFN1/PZFN2 were specifically designed to target exon 2 of porcine *MSTN* gene (Fig. 1A, Supplementary figure S1). The specific cleavage RSRR site for producing the N-terminal propeptide and the C-terminal mature myostatin is located 51bp downstream of the target site. Modification of the specific ZFN target sites is expected to result in loss of MSTN function. Following co-transfection with PZFN1 and PZFN2 plasmids to Ms primary fetal fibroblasts, PCR products of mixed cells were cloned to TA vectors and sequenced to determine targeting efficiency and types of mutations. The targeting efficiency of this ZFNs is ~4% when transfected into primary porcine fetal fibroblasts. Single-cell colonies were produced by limiting dilution in drug-free cell culture medium. We screened 1800 cell colonies using sequencing method. Of these 1800 cell colonies, 80 *MSTN*-mutant cell colonies were identified. 80 *MSTN-*mutant cell colonies including 78 heterozygous mutation (*MSTN*^+/−^) and 2 homozygous mutation (*MSTN*^−/−^) were identified by DNA sequence analysis (Fig. 1B). Over 90% of these mutants were short fragment deletions or insertions (<20 bp) (Fig. 1C). Of these 80 *MSTN-*mutant cell colonies, fourteen were selected as donor cells to produce *MSTN-*mutant piglets by using SCNT (Fig. 1D). Please note that cell colony #172 which is a *MSTN* homozygous mutant, did not generate any live offsprings (Supplementary Table S1). A total of 19 mutant piglets were born live from cell colony #105, #110 and #127 (Supplementary Table S1). Among these mutant piglets, 8 piglets from cell colony #105 and 1 piglet from cell colony #110 grew to adulthood. Colony #105 had a 15 bp deletion and a T-G mutation in one allele, and colony #110 had an 11 bp deletion in one allele. DNA sequencing confirmed that these nine piglets have the same mutations in *MSTN* as their donor cell colonies. The cloned male F0 *MSTN*^+/−^ pigs from colony #105 were mated with WT pigs to generate F1 female and male *MSTN*^+/−^ pigs. F1 heterozygous pigs were then bred to generate F2 *MSTN*^−/−^ , *MSTN*^+/−^ and *MSTN*^+/+^ pigs (Fig. 1E), with a ratio of 1:2:1 for these three genotypes (*MSTN*^−/−^: *MSTN*^+/−^: *MSTN*^+/+^), conforming to the Mendel’s law of inheritance.

In addition, FokI nuclease, CMV, and KanR domain was amplified by PCR (Supplementary Table S2) to examine whether ZFN plasmid DNA had integrated into the porcine genome. No integration of ZFN plasmid was detected in the DNA from all piglets tested (Supplementary Fig. S2). Sequence analysis of 11 predicted off-target sites indicated that there were no off-target cleavage events observed (Supplementary Tables S3 and S4).

### Expressions of *MSTN* mRNA and protein in *MSTN*-mutant Ms pigs

**Figure 2:**
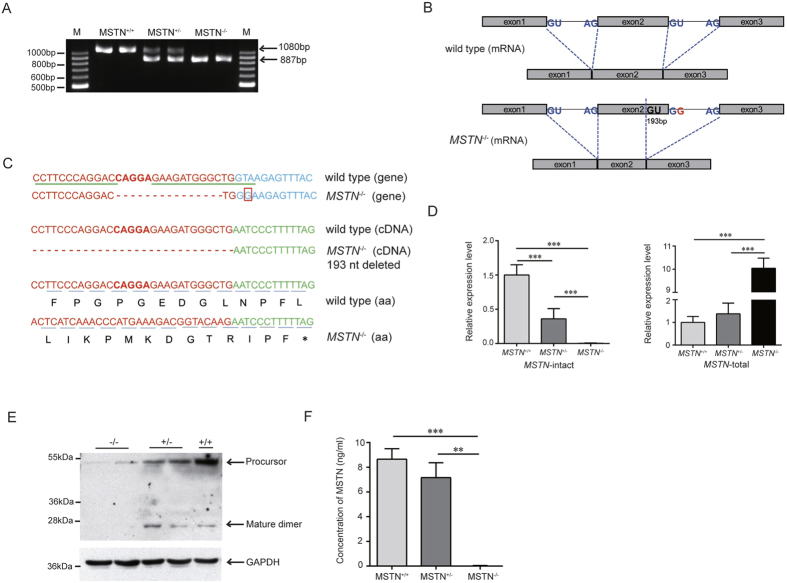
Changes of *MSTN* mRNA and protein in *MSTN* mutant Ms pigs. (**A**) Agarose gel electrophoresis of RT-PCR products derived from the coding sequence of *MSTN* mRNA. A single band was obtained in *MSTN*^+/+^ and *MSTN*^−/−^ pigs respectively, but there is approximate 200 bp difference in size between these two bands. Two bands were obtained *MSTN*^+/−^ pigs. (**B**) Change in splicing of *MSTN* mRNA exons after a T-G mutation. (**C**) Sequencing of the RT-PCR products indicated that *MSTN* gene from *MSTN*^−/−^ pigs had a T-G mutation at the beginning of intron 2 except for a 15-bp deletion, which result in intron error splicing—the 3’end of exon 2, resulting in a 193-nt deletion. The altered splicing of RNA caused frameshift, resulting in a premature termination of translation. Red, blue, and green letters represent the sequences of exon 2, intron 2, and exon 3 of *MSTN* gene respectively. Green underline: ZFN-targeted site; red box: single nucleotide mutation; and asterisk indicates the stop codon. (**D**) Real time quantitative PCR results of *MSTN*. Total RNAs were isolated from *longissimus dorsi* of *MSTN*^+/+^, *MSTN*^+/−^ and *MSTN*^−/−^ pigs. The expression levels were analyzed using the ΔΔCt method and normalized against GAPDH. Each sample was run in triplicate. (**E**) Detection of MSTN protein in skeletal muscle (*longissimus dorsi*) by Western blot. Protein extracts (20 μg) from of 8-month-old male pigs were subjected to SDS-polyacrylamide gel electrophoresis, blotted and probed with anti-MSTN antibody. Precursor (52 kDa) and mature dimer (26 kDa) were indicated by arrow. GAPDH protein was used as an internal reference to demonstrate equal amounts of proteins were loaded. (**F**) ELISA analysis of MSTN protein in porcine serum. Mature MSTN protein wasn’t detected in *MSTN*^−/−^ pigs using an antibody recognizing the C-terminal domain of MSTN protein, and the level of MSTN protein in *MSTN*^+/−^ serum decreased compared with *MSTN*^+/+^ serum. The error bars represent the standard error of the mean. *P < 0.05, **P < 0.01, ***P < 0.001, Student’s t test.

To detect the impact of the *MSTN* gene mutations on *MSTN* mRNA expression, we extracted total RNA from *longissimus dorsi* of *MSTN*^+/+^, *MSTN*^+/−^ and *MSTN*^−/−^ pigs, produced from colony #105, at the age of 8 months, then the coding sequence of *MSTN* mRNA was amplified and sequenced (Fig. 2A). In the reverse transcriptase-PCR, it is clear that *MSTN*^−/−^ homozygote only have a small PCR fragment compared with the wild-type, which had a big PCR product. In *MSTN*^+/−^, there are two PCR products. Both PCR fragments were cloned to the TA vector and sequencing analysis of the two PCR products indicate that the small fragment is 887 bp and the big fragment is 1080 bp. The T-G mutation derived from colony #105 disrupted the normal splicing of RNA, resulting in a 193-nt deletion in exon 2 (Fig. 2B). The altered RNA splicing caused a frameshift and premature termination of translation, resulting in possible production of a peptide of 188aa (Fig. 2C). The transcription level of *MSTN* was measured via real-time PCR (qRT-PCR). To detect intact *MSTN* mRNA levels, we designed two sets of primers to amplify *MSTN* mRNA. The primer pairs used for detecting *MSTN*-total are located in Exon1 region (1546–1565) and the beginning region of Exon2 (3534–3552) respectively, and the primer pairs used for detecting *MSTN*-intact are located in the end region of Exon2 (3744–3766) and Exon3 (5903–5922). The detailed information about the primer sequences and *MSTN* genomic DNA sequences were reported in Supplementary Fig. S1. Since the segment with 193-nt deletion in mRNA is located 3605–3798, the primer set of *MSTN*-intact would not be able to amplify any fragment with mRNA samples from *MSTN*-mutant pigs. The results demonstrated that the mRNA sample from wild-type pigs can be successfully amplified by the primer sets of the *MSTN*-total and *MSTN*-intact. However, there was no PCR amplification with mRNA samples from *MSTN*^−/−^ pigs with the primer set of *MSTN-*intact. The level of intact *MSTN* mRNA in heterozygous individuals decreased compared with WT pigs (Fig. 2D). The results with the primer set for *MSTN*-total showed successful amplifications for all pigs. The qPCR results indicated that total *MSTN* mRNA in *MSTN*^−/−^ pigs was substantially greater than in *MSTN*^+/+^ pigs, the amplified products apparently are from mutant mRNA since the intact mRNA were not detected. The length of amplified DNA fragments is shorter in *MSTN*^−/−^ pigs than that in wild-type pigs. The transcription level detected by these primers in *MSTN*^−/−^ pigs suggest that mutant *MSTN* gene can be transcripted to RNA in *MSTN*^−/−^ pigs, but can not be successfully translated to functional myostatin protein due to the 193-bp deletion in Exon2. As a result of the defects in *MSTN* translation, feedback signal may enhance the transcription and accumulate more products of mutant *MSTN* mRNA. The results from Western blotting showed MSTN precursor (52 kDa) and C-terminal mature myostatin dimer (26 kDa) bands in skeletal muscle extracts from the wild-type pigs and heterozygote (*MSTN*^+/−^), but both protein bands were not detectable in *MSTN*^−/−^ pigs (Fig. 2E). We also employed ELISA to detect myostatin protein in serum. Serum concentration of mature myostatin is 8 ng/ml and 6 ng/ml in wild-type and *MSTN*^+/−^ pigs, respectively, and it was not detectable in the serum of *MSTN*^−/−^ pigs (Fig. 2F). Therefore the results from Western blotting and ELISA confirmed that there was no functional MSTN protein in *MSTN*^−/−^ Ms pigs.

### Double-muscled phenotype of *MSTN* ^−/−^ Ms pigs

**Figure 3:**
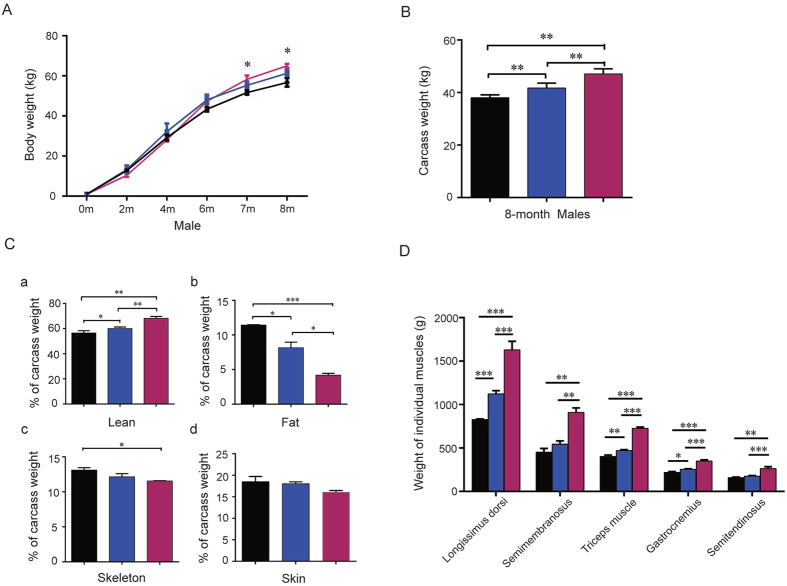
The increased body weight results from an increase in muscle mass in of *MSTN* mutant Ms pigs. (**A**) Changes in average body weight of *MSTN*^+/+^, *MSTN*^+/−^ and *MSTN*^−/−^ pigs from F2 sib or half-sib families at different ages (n = 3–6). (**B**) Average carcass weight of F2 8-month-old male pigs (n = 3–6). (**C**) Relative percentage of lean (a), fat (b), skeleton (c) and skin (d) of carcass weight in 8-month-old male pigs. (**D**) Average weight of individual skeletal muscles which were dissected on one side of the body. Black bar: *MSTN*^+/+^; blue bar: *MSTN*^+/−^; red bar: *MSTN*^−/−^. Samples were collected from 8-month-old male pigs. Data are expressed as mean  ±  SEM. *P < 0.05, **P < 0.01, ***P < 0.001, Student’s t test.

The body structure of *MSTN*^−/−^ pig appears distinguishable from *MSTN*^+/−^ and *MSTN*^+/+^ pig, with obvious wide hips and backs. *MSTN*^−/−^ pigs have similar phenotypic characteristics of the DM beef cattle body structure (Fig. 1E). There were not significant differences in body weight in the early growth stage up to 6 months of age among three genotype pigs. However, the average body weight of male *MSTN*^−/−^ pigs was heavier than *MSTN*^+/+^ pigs after 6 months of age. At the age of 8 months, the average body weight of male *MSTN*^−/−^ pigs significantly increased by 14.6% compared to *MSTN*^+/+^ pigs (Fig. 3A). To confirm if the changes in body weight of *MSTN*^−/−^ pigs were caused by skeletal muscle mass, we evaluated the carcass of male pigs at 8-month-old. The average carcass weight of *MSTN*^−/−^ pigs was 47.07 ± 1.40 kg, significantly heavier by 23.77% than *MSTN*^+/+^ control pigs (38.03 ± 0.54 kg). There were significant differences (*P* < 0.01) in carcass weights between wild type (WT) and mutant pigs (*MSTN*^−/−^ and *MSTN*^+/−^), and *MSTN*^−/−^ carcass was significantly heavier than *MSTN*^+/−^ carcass *(P* < *0.01)* (Fig. 3B). Further analysis of muscle, fat, skeleton, and skin tissue indicated that the lean percentage was greater in *MSTN*^−/−^ pigs by 8.08% and 11.62%, respectively, than that in *MSTN*^+/−^ and *MSTN*^+/+^ pigs. On the other hand, the percentage of body fat is lower in *MSTN*^−/−^ pigs by 3.27% and 7.23%, respectively, than that in *MSTN*^+/−^ and *MSTN*^+/+^ pigs. Ratios of skeleton and skin to carcass weights decreased in *MSTN*^−/−^ pigs compared with *MSTN*^+/−^ and *MSTN*^+/+^ pigs (Fig. 3C), indicating that the increased carcass weights in *MSTN*^−/−^ pigs came from skeletal muscle mass. To further investigate the effects of targeted *MSTN* mutation on individual muscles, we dissected *longissimus dorsi*, *triceps*, *semitendinosus*, *semimembranosus*, and *gastrocnemius* from 8 month-old male pigs. The average weights of individual muscle in *MSTN*^−/−^ pigs increased significantly by 59.85% to 101.71% compared with *MSTN*^+/+^ pigs. Particularly, the weights of *semimembranosus* and *longissimus dorsi* increased by 101.71% and 96.67%, respectively (Fig. 3D, Supplementary Table S5). These dramatic increases in *semimembranosus* and *longissimus dorsi* are the major reasons why *MSTN*^−/−^ pigs have large hips and wide backs, important characteristics of DM beef cattle body structure.

### Increased muscle mass of *MSTN* ^−/−^ Ms pigs results from muscle fiber hyperplasia rather than hypertrophy

**Figure 4:**
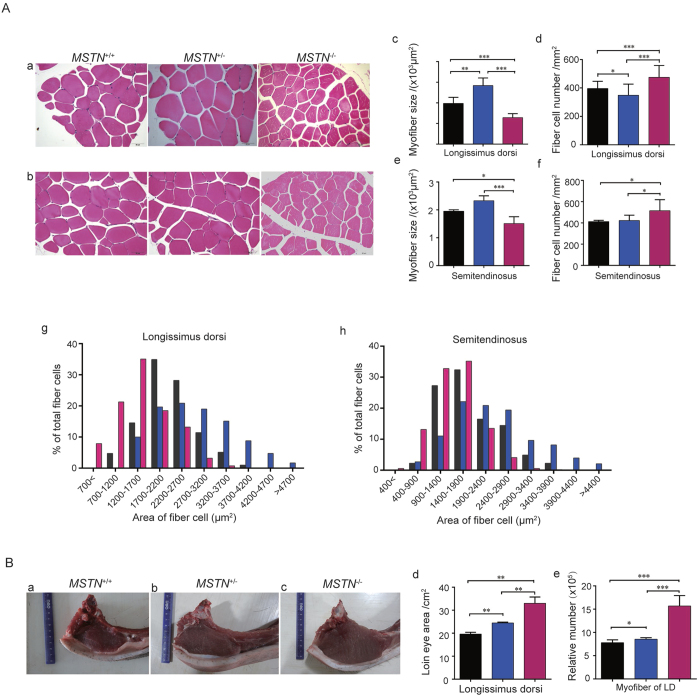
Increased muscle mass of *MSTN*^−/−^ Meishan (Ms) pigs is a result of hyperplasia rather than hypertrophy of muscle fibers. (**A**) Changes in myofiber size and density in *MSTN* editing Ms pigs. Histological cross section of *longissimus dorsi* (a) and *semitendinosus* (b). Scale bar = 50 μm. Average size and density of myofiber in *longissimus dorsi* (c,d) and *semitendinosus* (e,f). Distribution of different sizes of myofibers in *longissimus dorsi* (g) and *semitendinosus* (h) from *MSTN*^+/+^, *MSTN*^+/−^ and *MSTN*^−/−^ pigs. (**B**) Loin eye area and number of myofiber in *longissimus dorsi*. Photos of cross section of *longissimus dorsi* (called loin eye muscle) at the last rib (a–c); loin eye area of *longissimus dorsi* from *MSTN*^+/+^, *MSTN*^+/−^ and *MSTN*^−/−^ pigs (d); and relative number of myofiber in *longissimus dorsi* (LD) calculated from loin eye area and myofiber density (e). Black bar: *MSTN*^+/+^; blue bar: *MSTN*^+/−^; red bar: *MSTN*^−/−^. Samples were collected from 8-month-old male pigs. Data are expressed as mean ± SEM. *P < 0.05, **P < 0.01, ***P < 0.001, Student’s t test.

To determine if the increased muscle mass is due to hyperplasia and/or hypertrophy of muscle fibers, *longissimus dorsi* and *semitendinosus* were examined histologically (Fig. 4A). The average size of myofibers in *longissimus dorsi* from *MSTN*^−/−^ pigs (1286.01 ± 77.36 μm^2^) was substantially smaller than in *MSTN*^+/+^ pigs (1975.51 ± 111.20 μm^2^). On the other hand, the average size of myofibers in *MSTN*^+/−^ pigs (2837.31 ± 214.41 μm^2^) increased in comparison with *MSTN*^+/+^ pigs (Fig. 4A). Distribution of different sizes of myofibers showed that the relative percentage of smaller fiber cells in *MSTN*^−/−^ pigs is greater than in *MSTN*^+/+^ pigs, while the relative percentage of larger fiber cells in *MSTN*^+/−^ pigs is higher than in *MSTN*^+/+^ pigs (Fig. 4A). Corresponding to these observations in cell size distribution, the average density of myofibers in *longissimus dorsi* from *MSTN*^−/−^ pigs (475.25 ± 18.13/mm^2^) was significantly higher than in *MSTN*^+/+^ pigs (396.35 ± 10.51/mm^2^). However, the average density of myofibers in *MSTN*^+/−^ pigs is 348.88 ± 15.57/mm^2^, lower than in *MSTN*^+/+^ and *MSTN*^−/−^ pigs (Fig. 4A). The average size of myofibers in *semitendinosus* from *MSTN*^−/−^ pigs (1508 ± 93.33 μm^2^) was significantly smaller than in *MSTN*^+/+^ pigs (1954 ± 34.99 μm^2^) and the average density of myofibers in *semitendinosus* from *MSTN*^−/−^ pigs (514.8 ± 39.47/mm^2^) was significantly higher than in *MSTN*^+/+^ pigs (412.9 ± 4.36/mm^2^) (Fig. 4A), although the difference is not at the same magnitude as observed for *longissimus dorsi*. Additionally, the degree of difference in cell size distribution of myofibers in *semitendinosus* is also less significant than observed in *longissimus dorsi* (Fig. 4A). These results are consistent with what were observed for relative percentage of individual muscles. For example, when compared with *MSTN*^+/+^ pigs, the average weight of *longissimus dorsi* in *MSTN*^−/−^ pigs increased by 96.67% while the average weight of *semitendinosu*s in *MSTN*^−/−^ pigs increased by 64.52% (Table S5). The relative numbers of myofibers were calculated from the loin eye area and density of myofibers for *longissimus dorsi* (which has a greater increase in mass and regular cellular shape). At the age of 8 months, the loin eye areas of *longissimus dorsi* are 33.02 ± 2.72 cm^2^, 24.46 ± 0.35 cm^2^, and 19.66 ± 0.76 cm^2^, respectively, in *MSTN*^+/+^, *MSTN*^+/−^ and *MSTN*^−/−^ pigs (Fig. 4B). The calculated relative number of myofibers is 1.57 × 10^6^ in *MSTN*^−/−^ Ms pigs which is significantly greater than that in *MSTN*^+/−^ (8.54 × 10^5^) and *MSTN*^+/+^ (7.79 × 10^5^) pigs (Fig. 4B). These results clearly reflect the differences in the number of myofibers among three genotypes of *MSTN* pigs. Thus, we conclude that the increased muscle mass in *MSTN*^−/−^ Ms pigs primarily results from hyperplasia rather than hypertrophy of individual muscle fibers. In the meantime, the increased muscle mass of *MSTN*^+/−^ Ms pigs appears to result from fiber hyperplasia and hypertrophy.

### Effect of targeted *MSTN* mutation on axial skeletal patterning in Ms pigs

**Figure 5:**
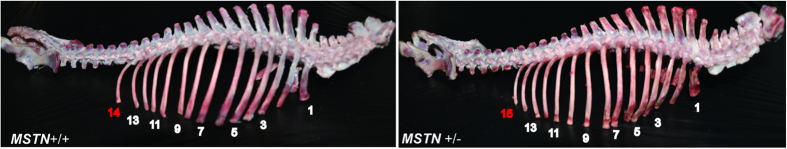
Skeletal patterns in wild-type and *MSTN* mutant Meishan (Ms) pigs. An example of one *MSTN* mutant pig (right) that contains fifteen thoracic vertebrae and one WT pig (left) that commonly contains fourteen thoracic vertebrae.

A closely related TGF-β family member to myostatin, namely GDF11, has been known to regulate axial skeletal patterning in knock-out mice. To study if there were any effects of targeted *MSTN* mutation on skeletal development, we counted the number of vertebrae. Statistical analysis of thoracic and lumbar vertebrae in all three genotype Ms pigs, including 59 *MSTN*^+/+^, 71 *MSTN*^+/−^ and 5 *MSTN*^−/−^, showed that the percentage of pigs containing fourteen thoracic and five lumbar vertebrae (T14L5) is 76.3% in *MSTN*^+/+^, 63.4% in *MSTN*^+/−^, and 60% in *MSTN*^−/−^ pigs, respectively; the percentage of pigs containing fourteen thoracic and six lumbar vertebrae (T14L6) is 23.7% in *MSTN*^+/+^, 16.9% in *MSTN*^+/−^, and 20% in *MSTN*^−/−^ pigs, respectively; and the percentage of pigs containing fifteen thoracic and five lumbar vertebrae (T15L5) is 0% in *MSTN*^+/+^, 19.7% *MSTN*^+/−^, and 20% in *MSTN*^−/−^ pigs, respectively (Fig. 5 and Table 1). Apparently, vertebrae pattern T15L5 is not present in the wild-type pig population. T15L5 with an extra thoracic vertebra is likely the consequence of targeted myostatin mutation in Meishan pigs since approximately 20% *MSTN*-mutant pigs (19.7% in *MSTN*^+/−^, and 20% in *MSTN*^−/−^) (Table 1). These results imply that myostatin also plays a regulatory role in the development and formation of thoracic vertebrae.

### Analysis of meat quality in wild-type and *MSTN* mutant Ms pigs

To investigate the effect of loss-of-function mutation in *MSTN* on meat quality, we measured several parameters such as pH, color, drip loss, cooking holding percentage (CHP), and tenderness. Results indicated that there are no obvious differences noted in meat quality parameters between *MSTN* mutant pigs and WT pigs except that the “a” value of meat color and shear force in *MSTN*^−/−^ pigs decreased compared to *MSTN*^+/+^ pigs (Table 2). Additionally, the main nutrition components (including protein, fat, moisture and amino acid) of *longissimus dorsi* from 8-month male Ms pigs indicated that there were no differences in total proteins and amino acids between three genotypes of pigs. However, the total fat in *MSTN*^−/−^ pigs decreased significantly compared to *MSTN*^+/+^ pigs (Supplementary Table S6), which is consistent with relative percentage of body fat in carcass (Fig. 3C). These results further confirmed that loss of myostatin function via targeted myostatin mutation have negative effects on body fat accumulation, including fat deposition in skeletal muscles.

### Evaluation of health status on *MSTN* mutant Ms pigs

To address the effect of targeted *MSTN* mutation on pig health, we analyzed hematological and biochemical parameters in 8-month-old male Ms pigs. There were no differences in hematology characteristics (Supplementary Table S7) and biochemical parameters among all three genotypes, except that the levels of serum creatinine (CR), glucose (GLU) increased while the triglyceride (TG) decreased in *MSTN*^−/−^ Ms pigs compared with *MSTN*^+/+^ pigs (Supplementary Table S8). Serum creatinine is 125.60 ± 14.17 μmol/L in *MSTN*^+/+^ pigs, 117.90 ± 11.79 μmol/L in *MSTN*^+/−^pigs and 209.50 ± 12.50 μmol/L in *MSTN*^−/−^ pigs. The higher CR level in *MSTN*^−/−^ Ms pigs than wild-type pigs is certainly due to the increased muscle mass. The increased serum creatinine level has been reported in DM cattle^33^. There has been reports that serum glucose level in MSTN mutant mice is lower than WT mice^23^. But it is interesting to note that serum glucose level is greater in *MSTN*^−/−^ Ms pigs than that in *MSTN*^+/+^ pigs at age of 8 months old. Further studies are required to investigate this phenomenon in pigs. The decreased serum TG level in *MSTN*^−/−^ Ms pigs is consistent with their lower percentage of body fat than wild-type Ms pigs.

## Discussion

High lean meat yield combined with low body fat has long been one of the ultimate goals in the breeding strategy of livestock industry^34^. In this study, we successfully generated *MSTN* loss-of-function mutant pigs by specifically targeting the exon 2 site of porcine *MSTN* gene with ZFN technology and somatic cell nucleus transfer. The percentage of lean meat yield of *MSTN*^−/−^ pigs at 8 months of age is 67%, approximately 12% greater than the corresponding *MSTN*^+/+^ pigs. Although DM cattle such as Belgian Blue beef cattle have 20% more muscle mass on average, less bone, lower fat, the breed indeed has several disadvantages, in particular, the reproduction issues due to unusually heavy and bulky offspring and reduced reproduction tract^15^. On the other hand, *MSTN-*mutant pigs generated in this study are as healthy as normal littermate control pigs. They develop and grow normally where they are raised and fed with the same normal diets as control pigs. So far, these *MSTN*-mutant pigs also have the same normal fertility as WT pigs without any abnormal pregnancy and other reproduction problems. Thus, these *MSTN-*mutant pigs will have an obvious advantage for the livestock meat industry. In addition, Meishan pigs are considered a famous Chinese pig breed for super-prolificacy because of its large litter size^30^. Like most of local Chinese breeds, they grow slowly and deposit huge amounts of body fat mass with low feed efficiency^35^. The dramatic increase in skeletal muscle mass in *MSTN*-mutant Meishan pigs demonstrate a way of fast genetic improvement for local fat-type indigenous pig breeds using gene targeting technology. Although the animals were generated by genetic manipulations via somatic cell nuclear transfer, the *MSTN*-mutant pigs do not have any foreign DNA and associated protein as a result of DNA sequence deletion by ZFN. To date, there is not any genetically modified (GM) livestock for food production^36^. The safety of the GM food is an important concern for consumers if the pig is used for commercial pig productions. It is notable that ZFN-edited *MSTN-*mutant pigs generated in our current study are very similar to those naturally occurring loss-of-function *MSTN* mutations observed in DM cattle. The pork from *MSTN*-mutant pigs will be as safe as the *MSTN*-mutant beef such as Belgian Blue, an important and popular beef cattle breed in the commercial beef production. Therefore, our ZFN-edited *MSTN-*mutant pigs should be safe to enter into commercial food supply following the review and approval process by government regulatory agencies.

The increase in muscle size in *MSTN*^+/−^ and *MSTN*^−/−^ mutant mice compared to wild type mice is due to fiber hyperplasia and hypertrophy of skeletal muscle fibers^6^. In our study, both hypertrophy and hyperplasia of skeletal muscle fibers at 8 months of age were observed in *MSTN*^+/−^ Ms pigs, but only fiber hyperplasia was observed in *MSTN*^−/−^ Ms pigs, which is consistent with the observation in DM cattle^33^,^37^. MSTN is an essential regulator of proliferation and differentiation of muscle cells during muscle development, which is detected from early myogenetic stage during embryonic myotome formation to adult skeletal muscle developmental stage^1^,^37^,^38^,^39^,^40^. Studies on muscle development demonstrated that muscle fiber number is primarily determined before birth, and the diameter of myofibers expands after birth^41^,^42^,^43^. The different patterns of myofibers observed in *MSTN*^+/−^ and *MSTN*^−/−^ are likely related to time of myofiber formation and postnatal muscle hypertrophy in *MSTN*-mutant pigs. Loss of MSTN functions can lead to an increase in the number of myofibers during embryonic phase and then an increase in diameter of myofibers after birth^38^. We believe that the number of myofibers increases significantly during embryonic phase in *MSTN*^−/−^ pigs, the significantly increased number of fibers may limit the enlargement or hypertrophy of all the myofiber after birth in the *MSTN*^−/−^ pigs with complete loss of myostatin function. In *MSTN*^+/−^ pigs, the function of MSTN gene is only partially lost, or with one copy of functional MSTN gene. The number of myofibers increases to some degree during embryonic phase, the magnitude is much lower than in *MSTN*^−/−^ pigs. During postnatal development, the diameter of myofibers in *MSTN*^+/−^ pigs can increase to some degree after birth compared with *MSTN*^+/+^ pigs, there resulting in muscle phenotype of fiber hyperplasia and hypertrophy.

Interestingly, the results from vertebrae counting showed an additional thoracic vertebra (T15L5, Fig. 5) in about 20% of *MSTN-*mutant pigs, which was not seen in the wild-type littermate control Ms pigs. To our knowledge, this phenomena was not observed or have not been studied in either *MSTN* knockout mice or other *MSTN-*mutant animals. Although the number vertebrae in most mammals are fixed at 19, swine appears variable due to genetic selection for meat production^44^,^45^. Wild boars have 19 vertebrae while genetically selected improved breeds have vertebrae numbers from 20 to 23^46^. In the wild-type control Meishan pig population (n = 59), 76% of pigs have 19 (T14L5) thoracic and lumbar vertebrae, 24% of pigs have 20 (T14L6) thoracic and lumbar vertebrae. However, the *MSNT* mutant pig population (n = 76), approximately 60% of pigs have 19 vertebrae, and 40% of pigs have 20 vertebrae. Although the effects of loss of myostatin function in the *MSTN*-mutant pigs did not dramatically change the number of vertebrae, it had an effect on skeletal formation during embryonic development. Recent studies demonstrated that MSTN and GDF11 have redundant functions in regulating skeletal patterning in mice^47^. *GDF11*^−/−^ mice contain more thoracic and lumbar vertebrae compared to *GDF11*^+/+^ mice. However, loss of both alleles of *MSTN* in *GDF11*^−/−^ mice resulted in a much more severe phenotype. Over-expression of transgene GDF-11 propeptide cDNA in bone tissues have resulted in transformation of the seventh cervical vertebra into a thoracic vertebra, and promote bone formation, and density in mice^48^,^49^. We also performed sequence analysis for the *MSTN*-mutant pigs (Supplementary Table S10), and did not observe any mutations in *GDF11* gene of *MSTN-*mutant pigs, implying that the T15L5 supernumerary thoracic vertebrae is the effect of targeted *MSTN* mutation not the GDF11 on skeletal development. Recent studies have showed the positive effects of myostatin inhibition on bone densities in *MSTN*-knockout mice through regulation of stem/progenitor cell potency^50^. Also, administration of soluble myostatin decoy receptor (ActRIIB-Fc) increased bone mass and bone volume fraction (BV/TV) significantly in the distal femur and lumbar vertebrae^51^. Clearly, there are animal experimental evidences that support positive effects of inhibition of myostatin function on skeletal formation and maintenances. Further investigations are required on molecular mechanisms of loss of myostatin function on skeletal formation and development, in particular the mechanism of MSTN and GDF11 redundancy in musculoskeletal formation and development. Regardless of the mechanism, there is a positive relationship between the number of thoracic vertebrae and pork yield. The increased number of thoracic vertebra has an economic benefit for pork production.

Meat quality analysis results indicated that there were no obvious differences between *MSTN* mutant pigs and WT pigs except that the “a” value of meat color and shear force in *MSTN*^−/−^ pigs compared to *MSTN*^+/+^ pigs, which is consistent with what was observed in DM cattle^42^. The “a” value of meat color is an indication of meat redness which reflects the level of oxymyoglobin. It has been known that the level of oxymyoglobin is higher in type I than in type IIB myofibers^52^. We speculate that a decrease in the “a” value in *MSTN*^−/−^ pigs indicates higher frequency of type IIB myofibers in *MSTN*^−/−^ pigs. We further analyzed *Myh4* protein (a specific gene of type IIB myofibers) in *longissimus dorsi* from *MSTN* mutant pigs. The results showed that the expression level of *Myh4* is significantly higher in *MSTN*^−/−^ pigs than in *MSTN*^+/+^ pigs (Supplementary Fig. S3), suggesting that *MSTN*^−/−^ pigs have more type IIB myofibers, which is consistent with the change of “a” value in *MSTN*^−/−^ pigs. The results of meat biochemical composition analysis indicated that there was no difference in total proteins and amino acids between three genotypes of pigs except the total fat. The decrease in muscle fat is consistent with decreased% fat in carcass. The value of shear force is an indication of meat tenderness, the smaller the shear force, more tenderer the meat. The decrease in shear force in *MSTN*^−/−^ pigs indicates the meat tenderness increases, which may be related to thinner myofibers in *MSTN*^−/−^ pigs. Most studies on DM cattle indicate that their meat tenderness is improved in comparison with normal (non-DM) beef cattle.

In conclusion, we have successfully generated *MSTN-*mutant Meishan pigs using ZFN technology in combination with somatic cell nucleus transfer. These *MSTN-*mutant pigs developed and grew normally, had increased growth performance after 6 months of age with dramatic skeletal muscle mass, producing a significant amount of carcass lean tissue and less body fat. These unique quality characters are particularly more apparent in *MSTN*^−/−^ pigs, showing great similarity in body structure to DM cattle with much higher muscle mass than *MSTN*-mutant heterozygotes and wild-type pigs. Moreover, one very interesting observation is that about 20% of *MSTN-*mutant pigs contain one extra thoracic vertebrae. This result provides us a new insight to better understand MSTN’s function in both skeletal and muscle formation and development in the future studies. The new *MSTN-*mutant pigs on Meishan pig genetic background generated in this study not only demonstrated a way of fast genetic improvement of lean-meat yield for local fat-type pig breeds, but also could serve as a great large animal model for biomedical studies in musculoskeletal development.

## Methods

### Ethics statement

All the *MSTN*^−/−^, *MSTN*^+/−^ and *MSTN*^+/+^ pigs were fed with the same standard diet and raised under the same conditions. All experimental protocols related to animal work described in this study were reviewed and approved by the Institutional Animal Care and Use Committee (IACUC) at Institute of Animal Sciences, Chinese Academy of Agricultural Sciences. All experiments were performed in accordance with the approved guidelines for animal care and management of research projects.

### Generation of zinc finger nucleases

Custom-made zinc finger nucleases (ZFN) plasmids designed for porcine *MSTN* gene were obtained from Sigma-Aldrich (St. Louis, MO) as shown in supplementary figure 1. The design and assembling of ZFNs were performed by Sigma-Aldrich as described elsewhere^53^. Measurement of ZFNs for gene disruption activity was performed by sequencing.

### Transfection, screening and identification of *MSTN* mutant cell line

All cell culture medium and reagents were purchased from Invitrogen. Primary porcine fetal fibroblasts were established from fetus collected from a 35 day pregnant Meishan sow and cultured in DMEM supplemented with 10% FBS, 10 mM MEM non-essential amino acids, 8 mM L-glutamine, 1 × sodium pyruvate, 1 × HT supplement, 2 ng/mL bFGF, and 1% Pen/Strep. For ZFN transfections, cells were transfected with the *MSTN*-ZFNs using Nucleofector^TM^ (AMAXA) according to the manufacturer’s instruction, with program T-016 being selected. After 48 h, cells were diluted and re-plated in 10 cm^2^ dishes (150 cells/dish on average). Positive colonies containing ZFN-mediated modification were screened and identified by DNA sequencing. Individual cell clones were isolated 11 or 12 days after culture, and then were expanded, sequence analyzed and cryopreserved after a total of 22–24 days in culture. Cells were incubated with 5% CO_2_ at 37 °C.

### Generation of *MSTN* mutant pigs by somatic cell nucleus transfer

Piglets were generated using somatic cell nucleus transfer with modified protocol named handmade cloning method (HMC) as described by Du *et al.*^54^. Fourteen different *MSTN*-mutant cell lines including one that contains a *MSTN* homozygous mutant, #172, were used as donor nuclei to generate the reconstructed embryos. The blastocysts at day 5 to day 7 with clearly visible inner cell mass produced from the HMC were surgically transferred to landrace sows on 5 or 6 days of standing estrus. Pregnancies were diagnosed by ultrasonography on day 28 after the surgery and confirmed every two weeks.

### Genotyping of *MSTN* mutants in Cloned piglets and offsprings

Genomic DNA was extracted from fetuses and newborn piglets and then was subjected to PCR (Supplementary Table S9, Supplementary Fig. S4). Following sequencing, the nucleotide sequence were analyzed via BioEdit to determine the genotype. “BioEdit” is a software for sequence editing and analysis. For heterozygote, PCR product of the targeted region of the *MSTN* gene was subcloned into the pGEM-TEasy Vector system (Promega) per manufacturer’s protocol, and twenty colonies for each sequence were picked and sequenced using the T7prom primer.

### RNA isolation and real-time RT-PCR

Total RNA was extracted using the Trizol reagent (Invitrogen), and cDNA was synthesized using the RevertAid TM First Strand cDNA Synthesis Kit (Fermentas) with 500 ng total RNA. The coding sequence of *MSTN* was amplified and sequenced. Each real-time PCR contained SYBR Select Master Mix (Life Technologies), 0.2 μM forward and reverse primers, 2 μL template cDNA, and dH_2_O up to a final volume of 20 μL. Reactions were performed on a 7500 FAST Real-Time PCR System (Applied Biosystems). Cycling conditions consisted of an initial, single cycle of 2 min at 95 °C followed by 40 cycles of 15 s at 95 °C and 1 min at 60 °C. Porcine *GAPDH* gene was used as an internal control to normalize the RT-PCR efficiency and to quantify the expression of the genes in WT pig, heterozygote- and homozygote *MSTN* mutant Ms pigs. All data were analyzed by the 2^−ΔΔCT^method using 7500 System SDS Software V1.4.0. RT-PCR primer for *MSTN* and real-time RT-PCR were shown in Supplementary Table S10.

### Western Blot and ELISA

Total proteins were extracted from *longissimus dorsi* using Thermo Scientific T-PER tissue protein extraction reagent (#78510). Protein concentration was measured with Micro BCA Protein Assay Kit from Thermo Scientific (#23235). Equal amount of total protein from *MSTN*^+/+^, *MSTN*^+/−^ and *MSTN*^−/−^ samples were loaded and separated by 12% SDS-PAGE. Following transfer of protein from gel to PVDF membrane, Western blot was performed using standard method. The primary antibodies against *MSTN* are from Abcam (ab55106) diluted at 1:1000. And the second antibodies are from CST (#7076) diluted at 1:2000. Color development was performed with SuperSignal West Pico Chemiluminescent Substrate from Thermo Scientific (#34080). Western blot for GAPDH (MBL, M171-7) was performed as an internal standard. The concentration of MSTN protein in serum was determined using an ELISA kit for porcine myostatin, with the antibody recognizing the C-terminal domain of MSTN protein (Bioss).

### Animal euthanasia, carcass dissection, and sample collection

Experimental pigs were euthanized, blood samples were collected. Hairs, heads, hoofs and internal organs were removed. Body weight, length, backfat thickness, and loin eye areas of carcass were measured, and the number of thoracic and lumbar vertebra were counted manually. Skin, bone, muscles and fat were then dissected from left carcass and their individual weights were determined. Total protein, total fat, moisture, amino acid, and essential amino acid were analyzed for *longissimus dorsi*. Tissue samples used for molecular detection were collected from right carcass and snap-frozen in liquid nitrogen.

### Histochemistry and analysis of myofiber size and number

Muscle samples for histochemistry were taken at the same location. After each tissue was fixed and slides prepared, haematoxylin and eosin staining was performed by a standard method. Slides were viewed by Olympus DP72 image system. For each sample, five view fields (areas) were randomly selected and then analyzed using IPP 6.0 software. The cross-sectional areas of 400–500 myofibers were measured, and average area of each myofiber, total number of myofibers and myofiber density were determined.

### Statistical analysis

Statistical comparisons of body weight, carcass character, myofibers and blood parameters among different genotypes of pigs were performed by the Student t test and p < 0.05 was considered as statistically significant. Statistical analyses were carried out using SAS release 8.1 (SAS Institute Inc., Cary, NC).

## Additional Information

**How to cite this article**: Qian, L. *et al.* Targeted mutations in *myostatin* by zinc-finger nucleases result in double-muscled phenotype in Meishan pigs. *Sci. Rep.* **5**, 14435; doi: 10.1038/srep14435 (2015).

## Supplementary Material

Supplementary Information

## Figures and Tables

**Table 1:** Skeletal analysis of *MSTN* ^+/+^, *MSTN* ^+/−^ and *MSTN* ^−/−^ Ms pigs generated from cell colony #105.

**Genotype**	Number of pigs dissected*	**No of pigs containing T14L5 (% of total)**	**No of pigs containing T14L6 (% of total)**	**No of pigs containing T15L5 (% of total)**
*MSTN*^+/+^	59	45 (76.3)	14 (23.7)	0 (0)
*MSTN*^+/−^	71	45 (63.4)	12 (16.9)	14 (19.7)
*MSTN*^−/−^	5	3 (60)	1 (20)	1 (20)

^*^Total number of pigs dissected is a summary of F1 and F2 pigs that were euthanized during experimental period. The percentage of each axial skeletal pattern is shown in parentheses. T: thoracic vertebra. L: lumbar vertebra.

**Table 2:** Meat quality traits of 8-month-old *MSTN* ^+/+^, *MSTN* ^+/−^ and *MSTN* ^−/−^ Ms pigs.

**Gene type**	**pH**		**Color in LD (45 min)**			**Drip loss/%**	**Shear force/kg. f**	**CHP/%**
	**pH (45 min)**	**pH (24 h)**	L*	a*	b*			
*MSTN*^+/+^	6.45 ± 0.13	6.25 ± 0.23	39.17 ± 1.08	11.18 ± 0.85	5.76 ± 0.45	1.03 ± 0.19	6.890 ± 0.03	65.85 ± 4.43
*MSTN*^+/−^	6.55 ± 0.08	6.11 ± 0.12	40.63 ± 0.59	11.07 ± 0.61	5.32 ± 0.32	1.22 ± 0.12	6.57 ± 0.28	66.08 ± 1.42
*MSTN*^−/−^	6.26 ± 0.11	5.50 ± 0.07	40.63 ± 0.59	5.56 ± 0.64#,&	4.03 ± 0.29	0.99 ± 0.10	5.55 ± 0.06^a^	71.94 ± 1.82

LD, *longissimus dorsi*. CHP, cooking holding percentage.

^#^Statistically significantly different between *MSTN*^−/−^ and *MSTN*^+/+^ pigs (p < 0.01).

^&^Statistically significantly different between *MSTN*^−/−^ and *MSTN*^+/−^ pigs (p < 0.01).
