# Supplementary material for: Targeted mutations in myostatin by zinc-finger nucleases result in double-muscled phenotype in Meishan pigs
Source: Sci Rep. 2015 Sep 24;5:14435. doi: 10.1038/srep14435 (PMC4585837; doi:10.1038/srep14435)
Supplement: Supplementary Information [file srep14435-s1.pdf]

SUPPLEMENTARY INFORMATION

Targeted mutations in *myostatin* by zinc-finger nucleases result in double-muscled phenotype in Meishan pigs

Lili Qian[1,2], Maoxue Tang[1], Jinzeng Yang[3], Qingqing Wang[1], Chunbo Cai[1,2], Shengwang Jiang[1], Hegang Li[1,4], Ke Jiang[4], Pengfei Gao[1], Dezun Ma[1], Yaoxing Chen[5], Xiaorong An[2] , Kui Li[1], Wentao Cui[1,*]

[1]Institute of Animal Sciences, Chinese Academy of Agricultural Sciences, Beijing 100193, P R China; [2]State Key Laboratory of Agrobiotechnology, China Agricultural University, Beijing 100193, PR China; [3]Dept of Human Nutrition, Food and Animal Sciences, University of Hawaii at Manoa, Honolulu, Hawaii 96822, USA; [4]Institute of Animal Sciences, Qingdao, 266100, P R China; [5]College of Animal Medicine, China Agricultural University, Beijing, 100193, P R China.

*Correspondence should be addressed to Wentao Cui (cuiwentao@caas.cn).

1 taaggttgag agtttgagct ctacagaggc cacttaaatt tagagaacaa aaagctctat
61 tctctgctcc cagaccttac cccaaatccc tgccaggtgt ctgccctctg gtcaaatgag
121 aaactggcaa aggggtgcaa acctagcaca gaattgggaa acagaaaaat gggcacccttt
181 tattatggtg ctccttctct tttatgtgtt tacaatactt gggcataatt tacagagaat
241 agatactaca ttttttactt tcaccactgg aaatctgagg caaactgcat tatcagtcat
301 aaaattcatt atctttctat tctaagttat tctaagctta ttctaagctc agatagctga
361 cattatcctc ttggtaataa acaatgaaaa aacacatctt ctgagcaata ttaatctgca
421 actttaggat aggaaaaaat cagttgraaa ctgagcacga ttttcacgtg aataaaagat
481 attatttaaa aataattcca tgtgtaatat aacagaataa gtatgatttt cattatgtac
541 tagaaattta gtcaggaaaa caagtttctc aaattatagc tgaatatatt ttactagtat
601 cacaatctta aattttaatt caggtcttcc taatttaaat ctgtatttct ctgattacac
661 aggactaaaa ataatttaaa acagcaaata aaattctttt ttcctcaaat gtttgtctaa
721 ataatgtaaa atcattttat ttttttgagg aaaaagacat ttcaactttt taagtatgaa
781 gtgtaaaaga attacttatt taaattacaa ttttaaagtt tcactaataa agattaataa
841 tatttaagtg cagtttatat tattgttaac atagatttwa atttttcaaa tgtcacatat
901 atctttcatt atttgtagat ttatttcttt tatgaagtag tcaaatgaat cagctcaccc
961 ttgactgtaa caaaatactg tttggtgact tgtgacagac agggttttaa cctctgacag
1021 cgagattcat tgtggagcaa gagccaatca tagatcctga cgacacttgt ctcatcaagt
1081 ggaatataaa aagccacttg gaatacagta taaaagattc actggtgtgg caagttgtct
1141 ctcagacagt gcaggcatta aaattttgct tggcgttact caaaagcaaa agtaaaagga
1201 agaaataaga acaaggagaa agattgtatt gattttaaaa tcatgcaaaa actgcaaatc
1261 tatgtttata tttacctgtt tatgctgatt gttgctggtc ccgtggatct gaatgagaac
1321 agcgagcaaa aggaaaatgt ggaaaaagag gggctgtgta atgcatgtat gtggagacaa
1381 aacactaaat cttcaagact agaagccata aaaattcaaa tcctcagtaa acttcgcctg
1441 gaaacagctc ctaacattag caaagatgct ataagacaac ttttgcccaa agctcctcca
1501 ctccgggaac tgattgatca gtacgatgtc cagagagatg acagcagtga tggctccttg
1561 gaagatgatg attatcacgc tacgacggaa acgatcatta ccatgcctac agagtgtaag
1621 tagtcctatt agtgtatatc aacaattctg ctgactgttg ttccagtgtt tatgagaaac
1681 agatctattt tcaggctctt ttaacaagct gttggcttgt acgtaagtag gaggaaaaag
1741 agtttctttt ttcaagattt catgagaaat aaactaatga gactgaaagc tgctgtatta
1801 ttgttttcct tagctaaaca gctgaaaata aaaaataaaa tgcttgcata gcattgttat
1861 atagtttaat aagacaaata taacatgctt atgctttcac agcttaatgc caccaaggca
1921 aggattggga gtttctacaa gcaatgtgga aaaaaaggtt ggttgtctga aataggcatt
1981 tgtaataaca ggtttttttc actaatgata aagaagggaa gatgtaaatt tgcagatatt
2041 gagccccatt ggggcatttg ctgcacccct agaatgactt ctgttattca gaacgatttc
2101 tcacagtgtt tctatgttct tcacaaatta aaatgtctaa ttttgaaagc tattacactg
2161 gaaagtataa aaaatatttt taaaaaattt aatgttttgg taagagcaat gatgaagtaa
2221 acatagcata atggtaatta tgagctaatt atcagaaaat gccaagaaat aaacatttta
2281 tcaagtaggt tatggctcac aaagtcctgc ttataccttg accatggtac tattgttgag
2341 agtaccctgt ctgaatattt ccaggcaggc acatgcttaa taagctctac aatattattt
2401 tctttttcat aggagggaga aagaactatt acctgtagta tccacattgc ttatgaagga
2461 caatatattt cataccattc ctattacaat cagttcaaaa gtatacacaa ggaaagggag
2521 acaggcacct taacagagaa ggcatgacaa gaaagatttt tgtgccatgt gtctgtgatt
2581 ttgctttata cagtgcttta cccactttaa actagactca aaacagtttc aaaatattat

2641 tcttcttatt aagtaattag gctataatgc aacaaataat ttttcttgaa aactatgcta
2701 tcagataatc ctagagtaga tttgccttat ttataaacaa tcttggaaaa ccaaaaggaa
2761 agctgtttct aaatgcttct gcttacaatg acagcatggc cttaacaatg ttttctaagt
2821 tttgagatag cctgaatgca acatttaaat tctggtgcta agtgccttct agtttggttc
2881 ctttaaaaaa gctatcccag gccaaaacat aacagatgta ctatattttc tactaattcc
2941 cgaggctcag ttagttgctc agtgtgtctc gtccccaggt aattcaggcc tgggggaagg
3001 gttccttctt ccagactgat tggtacagct gctcagtaag tgtaactact cagattccca
3061 aagaattcta agtggatgtt cctccacagt gtctcttgtt ctctctaatc atcatcattt
3121 taaaatttca tccactcttc attcctttac agaattttct ttagtctaca gttttctaga
3181 aaggaagtag gtttctcata aacagctgaa aaaacatatt gaaaaaaatc tgaaaagcta
3241 tagtaattat ttcatttgat atttttctga attatgaatg aaattctaca gtttttcatt
3301 ttaaaagact aaatatgcat gcactattcc aatagaaaaa aagctcactg attaatatga
3361 aggagtttgt tcatttttca tgaaacaatt tcaataactc ttttcttttt ttactcattt
3421 ttagctgatc ttctaatgca agtggaagga aaacccaaat gctgcttctt taaatttagc
3481 tctaaaatac aatacaataa agtagtaaag gcccaactgt ggatatatct gagacccgtc
3541 aagactccta caacagtgtt tgtgcaaatc ctgagactca tcaaacccat gaaagacggt
3601 acaaggtata ctggaatccg atctctgaaa cttgacatga acccaggcac tggtatttgg
3661 cagagcattg atgtgaagac agtgttgcaa aattggctca aacaacctga atccaactta
3721 ggcattgaaa tcaaagcttt agatgagaat ggtcatgatc ttgctgtaa**c cttcccagga**
3781 **ccaggagaag atgggctggt a**agagtttac tgaaaataac actcttaaaa tcttgttatg
3841 tttttattca taatgtgaat gagtagtagt ggaaaataac taccagtttc ctaagctaga
3901 caaaagtatc ttaccccaat ggtagccctg tacccaataa aagtaggtgt tcagtttcat
3961 atcctatgaa ataccctctt gatactttta ctttgcatga ggatttagaa gaaaaaagtt
4021 ttactataat ccttaactta ggaaattctt ttgaattgga aatgaaacac aaattgcttt
4081 tcattgatat gccatatgat tatatgaata aaacatgaaa tcttcatatt ggattctagt
4141 atatacccaa gtaaatattt tttccctaga agagtgccaa gtgtgttaaa acctttttggt
4201 ttaataaagc agaaaaaaat aaactctaaa aatcataatt aaaaatgaaa tgcttttatt
4261 tatagcaatt aactacaaca tgtttagact tacatactat taaatataat atatttaaga
4321 tcccctcatg ataaatatgt tcattatttt gtaggctgtt gatgcactaa tatgtatgta
4381 gattactttg tgaattgccc ttaataaaat ttaaaacttt aggctagtaa acctgtaaca
4441 ctcaacttag ttctgaacta tctcactatt cttttgcaag aatttactta ggtaatgcca
4501 actaatttat tccaaggcca aaaagatgac aatgtcttat atattataaa aactaataaa
4561 aaccatttta aaacctagta taaatttaaa ggtacttgct cttctggttc atctcttctt
4621 ttgtttactt ctgctttcaa aaacttattt attgtgacca tattctttac ttccatttat
4681 tgttataatt tataagatac tatacttgca agcaataaat gttatctttt tagcttttaa
4741 atggtctcat ttgaaaagaa tatataatta gtaagtcata gctactttaa ataaaaactt
4801 attctttaag agattaaaca cttctccaag tgatctgttt ttctttaatt aaaacgttat
4861 taactcccaa aatgatgtta ttgttttttt ataatcttaa ataccaataa ttaccaggtc
4921 tattttgatt ttgatacagg ataaaaacta ctattaatta cttaagaatg tgttcttttt
4981 tatatgtacc attttcatga tcaaagttgg tgatatgact gaggttttga ttattattaa
5041 acagatagtt aatatgatat attcctcatt tttccaaatg aaaggaaaaa tgtcttatat
5101 ggaggaaaag attggggcag ggggattagt aaattattac ttaaatatct gaataggagg
5161 atttttcaat gaaaggataa aggaagaatg attgtatcat ctgaatcttt ccctcccttt
5221 cctggagttt gtcctttcaa cccagtatac ctaccactcc cttcatcacc tactttccca

5281 ttacagtccc tatgtgttgg gtggtaacta ttttgttttg gtgttaatat ccaagtttcc
5341 cttaataaca cctagtgaat ggaggaagga tgagcatacc tacccatcag acatatttag
5401 ccaccatatt taatcaacaa gcatgaagaa aggaagctag cctctcccct tcctttcctc
5461 ctgcctctct ctctcttctc tgtcctcgct ccctttcttc ccatcaatat tttcagagca
5521 cctcttatgc gccaggcatt gggatactca aactggagga aacaagaaaa aaaaaaaaaa
5581 aaggcgaaga cctcagggaa atttatattg ctgctatatt tttttgagcc tagtgtaaat
5641 taaaattcct taatgctgtg ccttttaaaa acacaaataa gcaaaatagt ttatttcttc
5701 aacagttaaa tccttagggt aggaaagtga ttcaggatct attgctaact attaactctt
5761 ctttcatttt cacacagaat cccttttag aagtcaaggt aacagacaca ccaaaaagat
5821 ccaggagaga ttttggactc gactgtgatg agcactcaac agaatctcga tgctgtcgtt
5881 accctctaac tgtggatttt gaagcttttg gatgggactg gattattgca cccaaaagat
5941 ataaggccaa ttactgctct ggagagtgtg aatttgtatt tttacaaaaa taccctcaca
6001 ctcatcttgt gcaccaagca aaccccagag gttcagcagg cccctgctgt actcccacaa
6061 agatgtctcc aatcaatatg ctatatttta atggcaaaga acaaataata tatggaaaaa
6121 ttccagccat ggtagtagat cgctgtgggt gctcatgaga tttatatttg gttcattact
6181 tcctaaaaca tggaaggttt ttcccctcaa taattttgaa actgtgaaat tatgtaccac
6241 aggctacatg cctggagtat gctacagtca cttaagcaca agcgacagta tatgaactaa
6301 aagagagaat atatgcaatg gttggcattt aaccatcaaa acaaatcata caataaaaag
6361 tttttatgat ttccagagtt tttgaacgag gagatcaaat tccatttatg ttcatatata
6421 ttacaacata tgcaggtaaa tgaaagcaat tctccttgtg ttctggtgaa ttaaaggagt
6481 atgctttaaa gtctatttct ttacaatttt acttaatatt tacagaaaaa tctatatgta
6541 gtattgataa gatgtaggat tgttatatac cattatttga gtcatcctta aacacttgaa
6601 ttatattgta tgatagcata cttggtaaga gaagattcca caaaaatagg gatggtacac
6661 catatgcaag ttcccattcc tattctgatt gatacagtac attgacaatt catgccaatg
6721 gtgctaattc aataggctga atggctggtg ttatcaggtt tatcaaataa aagcattcag
6781 taaagtaatg gttctccttt cttcaggtgc attttcatac tctttcaaat ggagaatgga
6841 ttttctttaa tgaaagaaaa atcatttttc tagagctctg cattcaattc tgtagcatac
6901 ttggagaaac tgcatttaaa aggcagccaa aaagtattca tttttatcaa aatttcaaaa
6961 ttatagcctg cctttgcaac actgcagttt ttatgataaa atcatggcaa tgactgattc
7021 tatcaatatt gtataaaaag actttgaaac aattgcattt atataatatg taaacaatat
7081 tgttttgtaa ataaatgtct ccttttttat ttactttggt atatttttat gtaaggatat
7141 ttcaaattaa gtattaaggc acaaatacat gtcatgtgac agaaaagcaa atgcttatat
7201 ttcggagcaa attagctgat taaatagtgg tcttaaaact ccatatgcta atggttagat
7261 ggttatatta caatcatttt atattttttt acattattaa cattcactat ggattcatga
7321 tggctgtata atgtgaatgt gaaatttcaa tggtttactg tcaatgtatt caaatctcaa
7381 cgttccatta ttttaatact tataagtaag cataccaaaa tgatttaact caattatctg
7441 aaatcagaat aataaactga tgatatctta agaattgtta atttaatttt ataattcgat
7501 aatgaatata tttctccata tatttacttc tattttgtaa attaggattt tgttaatcaa
7561 atacattgta cttatgacta agtgaaatta tttcttacat ctaatgtgta gaaacartwt
7621 aagtta//

Supplementary Figure S1. Sus scrofa breed Meishan myostatin (*MSTN*) gene sequence (>gi|134244150|gb|EF490988.1) and ZFN targeted knockout site. Red letters stand for specific site targeted by PZFN1/PZFN2. Blue letters stand for ZFN binding sites. Yellow

highlighted sections stands for three exons of *MSTN* gene. Underline stand for the location of the primers of *MSTN*-total and *MSTN*-intact.

Supplementary Table S1. Summary of handmade cloning results using ZFN-mediated porcine fibroblast cells

Cloning date	Recipient	Donor cells	No. of transferred reconstructed embryos	Pregnant sows	Stillborn	Liveborn	Cloning efficiency (%)	Piglet ID number and birth weight (kg)
1/2/2012	317	G4	83	—	—	—	0	
	366	G4	103	—	—	—	0	
1/3/2012	269	35/84/166/141	96	—	—	—	0	
1/4/2012	172	G4	94	—	—	—	0	
	205	G4/84	94	—	—	—	0	
1/5/2012	287	113/115/84	89	—	—	—	0	
2/28/2012	283	105	93	—	—	—	0	
	606	105	79	Delivered	3	0	3.8	606-1~606-3△
	368	105	79	Delivered	2	8	12.6	105-1△,105-2△, 191-198 (08-1.2)
3/5/2012	256	110	99	Delivered	1	0	1.0	256-1△
	195	110	125	—	—	—	0	
3/6/2012	269	105	78	—	—	—	0	
	379	110	81	—	—	—	0	
	508	110	90	—	—	—	0	
5/2/12	613	110	84	Delivered	1	2	3.6	613-1△, T-261 (0.8), T-262 (0.9)

	363	110	84	—	—	—	0	
	203	217	70	Resorbed	—	—	0	
8/6/12	283	T2-186	87	Delivered	2	0	2.3	283-1△, 283-2△
8/8/12	585	110	123	Resorbed	—	—	0	
8/13/12	426	T2-186	109	—	—	—	0	
8/14/12	395	172	99	—	—	—	0	
8/15/12	477	127	115	—	—	—	0	
8/20/12	441	T2-186	131	—	—	—	0	
8/21/12	470	127	126	Delivered	1	4	3.2	470-1 (0.8), 470-2~470-4 (0.4-1.0)▼, 470-5△
8/22/12	638	110	114	Delivered	0	5	4.4	638-1 (0.9), 638-2~638-5 (0.8-1.1)▼
9/4/12	641	127	70	—	—	—	0	
	642	127	66	Resorbed	—	—	0	
9/5/12	367	104	70	—	—	—	0	
Total	n=28		2631	Pregnancy rate 10/28=35.7%	10 10/29=34.5%	19 19/29=65.5%	In average 29/2631=1.1%	

△ Body weights were not available for stillborn piglets. ▼These piglets died after birth

Supplementary Table S2. Primers used for analysis of ZFN plasmid DNA integration

	Primers (5’-3’)	Size of PCR Product	Tm	Integration (+)/Normal (-)
PZFN-Fok1-F	AAGCAGAAAGCCTGACGG	550bp	56 ℃	-
PZFN-Fok1-R	TTAGGAAAGGACAGTGGGAGT			
PZFN-CMV-F	TGGAGTTCCGCGTTACAT	365bp	58 ℃	-
PZFN-CMV-R	TGAGTCAAACCGCTATCCAC			
PZFN-KanR-F	GGTGCCCTGAATGAACTGC	515bp	58 ℃	-
PZFN-KanR-R	CGGGTAGCCAACGCTATGT			
β -actin-F	GCCAACCGTGAGAAGATG	396bp	58 ℃	-
β -actin-R	TGCAAGGAACACGGCTAA			

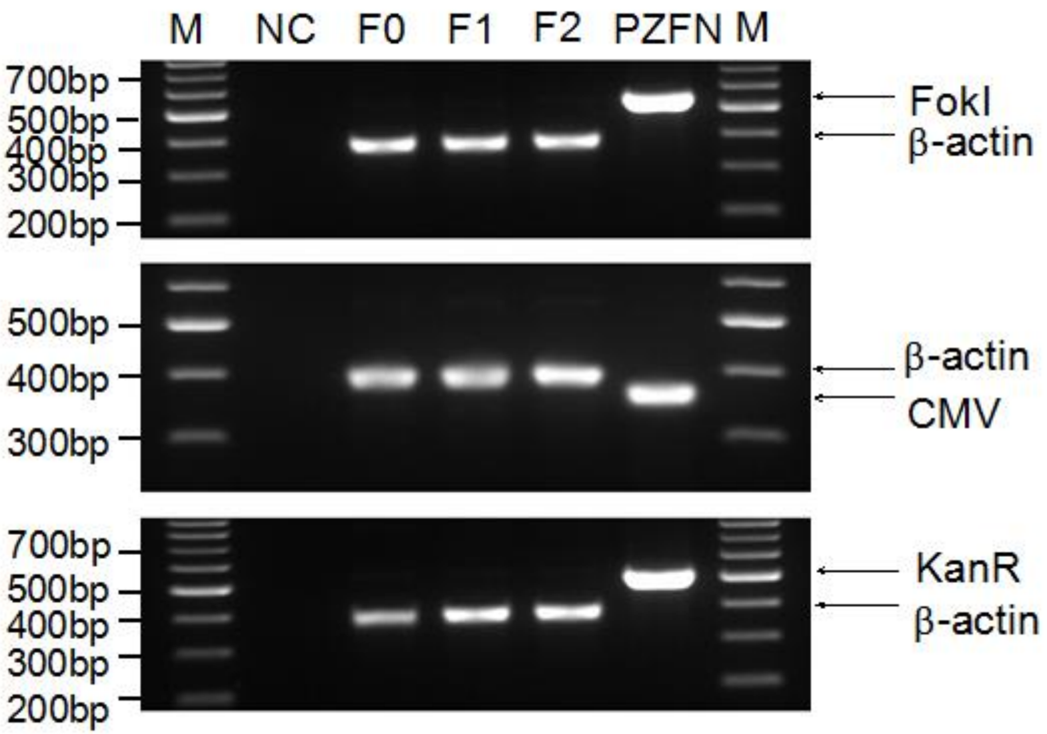

Supplementary Figure S2. PCR results of ZFN plasmid DNA in cloned piglets and their offsprings. F0, F1 and F2: PCR products from templates isolated from three generations of cloned founder piglets; PZFN: PCR product of ZFN-plasmid DNA; NC: H_2O; M: Marker. β-actin: PCR product of internal refence gene. None of the analyzed piglets shows FokI , CMV, or KanR integration.

Supplementary Table S3. Off-target analysis of cloned piglets generated from cell lines 105 and 110

Site #	Chr.	Location	Binding Sequence	No. Mismatches	Gene?	Off-target (+)/Normal (-)
1	chr7	5380790	CTACCtGCCCATCTTgGAGCGTcTCCTGGGAAaGC	4	KIAA1856	-
2	chr12	23216526	AtCTTCCCAGGAgAAGTGTGAAGgTGGGCTGGTtT	4		-
3	chrX	9196183	ACCaTaaCAGGACATAGGAGgAGATGGGCTtGTAA	5		-
4	chr2	90396700	ATACCAGCCCcTCTTCAACGCTGTCCTGaGAgacG	5		-
5	chr2	93650711	TtCTgCCCAGGACCACACTGAgGAgGGGCTGGTgT	5		-
6	chr14	1524938	CctCttGCaCATCTTCTGACCTGTCCTGGGAAGGC	5		-
7	chr14	117348558	TgAgCAGCCCAgCTTCCGGCAGGaCCTGGGAAGaG	5		-
8	chr13	15555955	TCtTTCCCAGGAgGGGGTGttGAaGGGCTGGTAG	5		-
9	chr16	30824552	CTACCtGtgCATCTTaGAGGGTcTCCTGGGAAGGC	5		-
10	chr4	2091602	CTAagtGCCCgTCcTCACCACAGTCCTGGGAAGGG	5	POLN	-
11	chr4	96457896	CTcCCAtCCCAcCTTCCTTTCGTCCTGGtAAaGG	5	UNC5C	-

These 11 highly homologous segments were analysed by sequencing PCR products.

Supplementary Table S4. Primers used for examining the off-targeting effect of the *MSTN*-specific ZFNs

Similar sequence location	Primer-F	Primer-R	Product size
rorochr7-5380790	AAAATAACTATGGCAGAGGCT	GTATCTGGAGGACTTTGGGT	469bp
chr12-23216526	TCAGCACATCGCTCCAAAT	GTCGTCCTGTCTCCAAGGTTAA	573bp
chrX-9196183	TTAGACTGTCTCAAATGGAAGG	ACTGTTCTGGGGCTTGTCT	479bp
chr2-90396700	AGCAGAAGGCAATGAGGC	CGAACTGGGTCTTGTGGG	465bp
chr2-93650711	CCTGGAGGCTTCTGTGAG	TGTGGGTCTGGAAGTGATG	538bp
chr14-1524938	GGCTTCCTTCCTTGTGCTAC	TGTTGGTGGCTTTGTGGC	511bp
chr14-117348558	CCCATCACTAAGAAAGGCC	CTGCCAGACGATCCACAT	528bp
chr13-15555955	TCTTTCTCAGGCAGGTTTAA	GCAAGGCACTGTGGTTCT	534bp
chr16-30824552	CTGGGAGTCTGGCATTCA	GGTGGAGCTGGGTCTTGT	517bp
chr4-2091602	TGTTTGTCAAGGCCCACG	GAACGAACGGCAACCAGA	481bp
chr4-96457896	CCATACCCTCTAACCCTTT	TCCTGGCAGATTCTTTCTT	574bp

Supplementary Table S5. Average weights of individual muscles from *MSTN*$^{-/-}$ and *MSTN*$^{+/+}$ pigs

	**Weights (g)**		**Percentage of *MSTN*$^{+/+}$**
	***MSTN*$^{+/+}$ (n = 4)**	***MSTN*$^{-/-}$ (n = 3)**	
Longissimus dorsi	828.30 ± 6.19	1629.00 ± 99.06	196.67
Semimembranosus	450.90 ± 44.01	909.50 ± 51.40	201.71
Triceps muscle	402.30 ± 17.05	725.00 ± 15.12	180.22
Gastrocnemius	218.90 ± 9.70	349.90 ± 13.46	159.84
Semitendinosus	159.80 ± 5.52	262.90 ± 21.47	164.52

Supplementary Table S6. Nutrition facts of longissimus dorsi from 8-month-old *MSTN*$^{+/+}$, *MSTN*$^{+/-}$ and *MSTN*$^{-/-}$ Ms pigs

**Name**	***MSTN***$^{+/+}$	***MSTN***$^{+/-}$	***MSTN***$^{-/-}$
Total protein, %	23.02±0.60	22.54±0.29	22.67±0.01
Total fat, %	1.10±0.16	1.04±0.14	0.61±0.04[a,b]
Moisture, %	74.20±0.70	75.18±0.40	75.07±0.32
Amino acid, %	22.23±0.41	21.94±0.31	22.48±1.15
EAA, %	8.23±0.22	8.19±0.13	8.405±0.53

There is no statistically significant difference in each nutrition ingredient among the *MSTN*$^{+/+}$, *MSTN*$^{+/-}$ and *MSTN*$^{-/-}$ pigs, except for the total fat, which is significantly decreased in *MSTN*$^{-/-}$ pigs compared with *MSTN*$^{+/+}$ and *MSTN*$^{+/-}$ pigs. [a]Statistically significantly different between *MSTN*$^{-/-}$ and *MSTN*$^{+/+}$ pigs ($p<0.05$), [b]Statistically significantly different between *MSTN*$^{-/-}$ and *MSTN*$^{+/-}$ pigs ($p < 0.05$). The “amino acid” including 17 of the 20 common amino acids, except for Trp, Gln and Asn. Essential amino acid (EAA) including 7 of the 8 essential amino-acid, except for Trp. Data are expressed as mean ± SEM.

Supplementary Table S7. Hematology characteristics of 8-month-old *MSTN*$^{+/+}$, *MSTN*$^{+/-}$ and *MSTN*$^{-/-}$ Ms pigs

**Parameter**	***MSTN*$^{+/+}$**	***MSTN*$^{+/-}$**	***MSTN*$^{-/-}$**
RBC (10^{12}/L)	7.72±0.11	7.74±0.54	7.31±0.54
HGB (g/L)	149.00±5.00	141.00±2.50	133.50±2.50
HCT (%)	47.70±2.80	45.20±2.15	42.75±2.15
MCV (fL)	61.50±2.50	58.45±1.40	58.60±1.40
MCH (pg)	19.30±0.40	18.25±1.05	18.35±1.05
MCHC (g/L)	313.00±8.00	312.00±10.00	313.00±10.00
WBC (10^{9}/L)	11.11±4.69	10.02±0.42	10.11±0.42
RDW-CV (%)	17.80±2.60	20.60±2.55	20.85±2.55
RDW-SD (fL)	37.25±0.25	37.75±4.30	39.00±4.30

There is no statistically significant difference in each parameter among the *MSTN*$^{+/+}$, *MSTN*$^{+/-}$ and *MSTN*$^{-/-}$ pigs.

Supplementary Table S8. Biochemical parameters of 8-month-old *MSTN*$^{+/+}$, *MSTN*$^{+/-}$ and *MSTN*$^{-/-}$ Ms pigs

**Parameter**	***MSTN*$^{+/+}$**	***MSTN*$^{+/-}$**	***MSTN*$^{-/-}$**
ALT (U/L)	41.40±2.54	36.00±2.75	39.00±3.00
AST (U/L)	113.00±15.76	138.20±22.41	90.50±5.50
TP (g/L)	76.46±2.50	79.01±1.15	76.90±5.80
ALB (g/L)	34.70±2.	33.16±1.27	32.15±2.85
GLOB (g/L)	41.76±1.38	45.85±1.73	44.75±2.95
UREA (mmol/L)	3.78±0.12	3.86±0.23	4.43±0.95
CR (μmol/L)	125.60±14.17	117.90±11.79	209.50±12.50[a,b]
GLU (mmol/L)	4.04±0.35	4.02±0.59	6.53±0.48[a]
TG (mmol/L)	0.14±0.02	0.10±0.01	0.05±0.01[a]
TC (mmol/L)	1.47±0.12	1.31±0.10	1.44±0.22
HDL-C (mmol/L)	0.56±0.08	0.45±0.06	0.68±0.16
LDL-C (mmol/L)	0.75±0.04	0.67±0.06	0.68±0.01
INS (pmol/L)	22.26±2.26	22.16±2.10	25.41±2.41

CR and GLU of *MSTN*$^{-/-}$ pigs are significantly increased compared with that of *MSTN*$^{+/+}$ pigs. TG of *MSTN*$^{-/-}$ pigs is significantly decreased compared with that of *MSTN*$^{+/+}$ pigs. There is no statistically significant difference in the other parameters among the *MSTN*$^{+/+}$, *MSTN*$^{+/-}$ and *MSTN*$^{-/-}$ pigs. [a]Statistically significantly different between *MSTN*$^{-/-}$ and *MSTN*$^{+/+}$ pigs ($p < 0.05$). [b]Statistically significantly different between *MSTN*$^{-/-}$ and *MSTN*$^{+/-}$ pigs ($p < 0.01$).

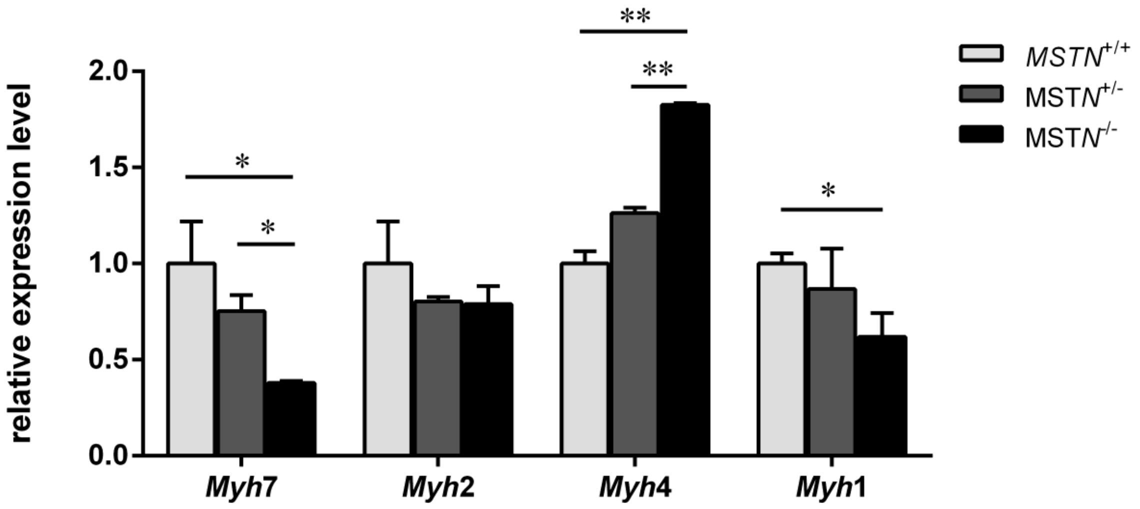

Supplementary Figure S3. Relative expression level of *Myh*7, *Myh*2, *Myh*4, *Myh*1 in longissimus dorsi from 8-month-old male *MSTN*$^{+/+}$, *MSTN*$^{+/-}$ and *MSTN*$^{-/-}$ Ms pigs. *Myh*7, *Myh*2, *Myh*4, *Myh*1 is the specific gene of type Ⅰ, type ⅡA, type ⅡB, and type ⅡX myofibers, respectively. The relative expression level of *Myh*4 (the specific gene of type ⅡB) is significantly higher in *MSTN*$^{-/-}$ pigs than in *MSTN*$^{+/-}$ and *MSTN*$^{+/+}$ pigs.

Supplementary Table S9. Primer pairs used for sequence analysis of the targeted region of *MSTN* gene

	**Primer pair sequences (5'-3')**	**Tm**	**Size of PCR Product**
PZFN1/PZFN2	TACAAGGTATACTGGAATCCGATCT<br>GCAAAGTAAAAGTATCAAGAGGGTA	58°C	397 bp

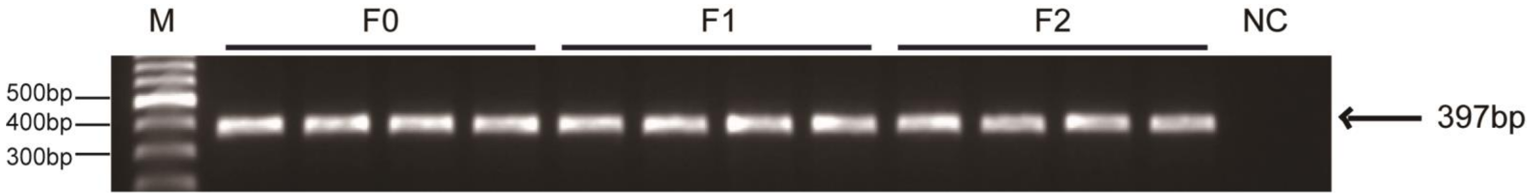

Supplementary Figure S4. PCR products of the targeted region of *MSTN* which were used for sequencing analysis of cloned piglets and their offsprings. F0, F1 and F2: three generations of cloned founder piglets; NC: H_2O; M: Marker (0.1 kb–1 kb in 0.1-kb steps).

Supplementary Table S10. Primers used for RT-PCR and real time quantitative PCR

Gene	Primer-F	Primer-R
MSTN（coding sequence）	ATGCTGATTGTTGCTGGTCC	CCCACAGCGATCTACTACCA
GDF11（coding sequence）	GTCATTAGCATGGCCCAGGA	ACTTGAGTGCTCATCGCAGT
MSTN-intact	TGAGAATGGTCATGATCTTGCTG	TCCAGTCCCATCCAAAAGCT
MSTN-total	AGTGATGGCTCCTTGGAAGA	TGTAGGAGTCTTGACGGGT
*Myh*7(fiber of type Ⅰ)	GCACTGAAGAGGCTGACAAG	ATCCGTGTCACCATCCAGTT
*Myh*2(fiber of type ⅡA)	CTGGCTGGTTGGACAAGAAC	GACACGGTCTGGAAGGAAGA
*Myh*4(fiber of type ⅡB)	TAGGAAGAAGCACGCAGACA	GGTTTCCCTTGGCTTTGGAG
*Myh*1(fiber of type ⅡX)	ATTTCTGACCTCACGGAGCA	ACCTCCGACTTGACTTGGTT
GAPDH	GTGAAGGTCGGAGTGAACG	CTCGCTCCTGGAAGATGGTG

Methods:

Analysis of Off-Target and ZFN Plasmid Integration into Porcine Genome

ZFN off-target sites were identified by ZFN manufacturer using bioinformatics tools. PCR primers were designed to flank the 11 most likely off-target sites (2 mismatches containing 4 bp and 9 mismatches containing 5bp) as detailed in Supplementary Table S3 and S4. These target regions were amplified in the knockout founder animals and the PCR products were directly analyzed for ZFN cleavage by sequencing. To test the possibility of ZFN plasmid integration into MSTN mutant piglets, Fok1 nuclease-specific PCR was performed using primers ZFN Fok1-F and ZFNFok1-R (Supplementary Table S2 and Fig. S2). PCR product of 550 bp length (obtained with an annealing temperature of 56 °C) was separated on an agarose gel (2%).

Hematology and blood biochemical analysis

Hematology was performed in a local hospital, including the following parameters: red blood cells (RBC), haemoglobin (HGB), hematocrit (HCT), mean corpuscular volume (MCV), mean corpuscular hemoglobin (MCH), mean corpuscular hemoglobin concentration (MCHC), White blood cells (WBC), red blood cell volume distribution width- coefficient of variation (RDW-CV), red blood cell volume distribution width-standard deviation (RDW-SD). Biochemical parameters were performed in Beijing CIC clinical laboratory using a biochemical analyzer (Vital Selectra E 2000, Holland). The following tests were performed: alanine aminotransferase (ALT), aspartate aminotransferase (AST), total protein(TP), albumin(ALB), globin(GLOB), urea, creatinine (CR), glucose (GLU), triglyceride (TG), total cholesterol (TC), high density lipoprotein cholesterol (HDL-C), low density lipoprotein cholesterol (LDL-C). Insulin (INS) was detected using an ELISA kit.
